# Effects of huoxin formula on the arterial functions of patients with coronary heart disease

**DOI:** 10.1080/13880209.2018.1561726

**Published:** 2019-06-14

**Authors:** Yan Xu, Hongyi Hu, Yi Li, Rong Cen, Chengzeng Yao, Wenhuan Ma, Minhua Huang, Yahui Yin, Hongzhi Gao, Yongming Liu, Alexander Endler

**Affiliations:** a Department of Cardiology, Shuguang Hospital Affiliated to Shanghai University of Traditional Chinese Medicine, Shanghai, China;; b Department of Gastroenterology, Longhua Hospital Affiliated to Shanghai University of Traditional Chinese Medicine, Shanghai, China;; c Department of Nephropathy, Shuguang Hospital Affiliated to Shanghai University of Traditional Chinese Medicine, Shanghai, China;; d Endoscopic Department, Shuguang Hospital Affiliated to Shanghai University of Traditional Chinese Medicine, Shanghai, China;; e Department of Cardiology, Zhabei District TCM Hospital, Shanghai, China;; f Department of Cardiology, Shanghai BaoShan District Combine Traditional Chinese and Western Medicine Hospital, Shanghai, China;; g Department of Molecular Medical Research, Tokyo Metropolitan Institute of Medical Science, Tokyo, Japan

**Keywords:** hsCRP, inflammatory factor, CAVI, ABI, IMT

## Abstract

**Context:** Huoxin formula is a Traditional Chinese Medicine for coronary heart disease (CHD) treatment.

**Objective:** To explore the therapeutic mechanism of the Huoxin formula on arterial functions in CHD patients.

**Materials and methods:** Fifty-eight CHD patients receiving cardiovascular drugs including β-receptor blocker, statins, and antiplatelet medications or others were randomized into intervention [additionally 13.5 g Huoxin formula granules dissolved in 150 mL warm water per time, twice a day (*n* = 30)] and control [only cardiovascular drugs (*n* = 28)] groups. Serum biomarkers (hs-CRP, IL-18, IL-17, TNF-α, MMP-9), and cardiovascular indicators of the common and internal carotid arteries (ICAs) were monitored before and after the treatments.

**Results:** After 3 months of treatment, the increases of intima-media thicknesses (IMT) of the left and right common carotid arteries (CCAs) as well as of the left and right ICAs and the increases of the left and right cardio-ankle vascular index were all significantly (all *p* < 0.001) less in the intervention than in control group (all *p* < 0.001). Serum concentrations reductions of hs-CRP, IL-18, IL-17 and MMP9 (all *p* < 0.001) levels were higher in the intervention compared to the control group, which correlated with the changes of left ICA (hs-CRP: *r* = 0.581, *p* = 0.009; IL-18: *r* = 0.594, *p* = 0.007; IL-17: *r* = 0.575, *p =* 0.006).

**Discussion and conclusion:** Since the Huoxin formula improved arterial functions and reduced inflammatory factor activities in CHD patients, a large-scale clinical trial is warranted.

## Introduction

Coronary heart disease (CHD) is characterized by coronary arterial stenosis or obstruction caused by progressive coronary atherosclerotic lesions, and the resulting myocardial ischemia or necrosis of the involved cardiac muscle (O'Flaherty et al. 2008; Hung et al. 2015). The onset of CHD is often characterized by coronary atherosclerosis (AS) and it is widely believed that AS results from the interaction of various risk factors (Duncan et al. 2003; Schmidt et al. 2005). Most researchers accept the ‘hypothesis of endothelium injury and reactions’ (Ross et al. 1977), which states that multiple risk factors damage the arterial endothelial cells and that consequent inflammatory-fibroplastic reactions result in the progressive development of atherosclerotic lesions (Duncan et al. 2003; Hadi et al. 2005). Symptoms of CHD are correlated not only with the extent of stenosis or occlusion, but also with the stability of the atheromatous plaques.

In recent years, accumulating evidence has shown that the generation of inflammatory factors is a key component that destabilizes the plaque. This evidence emphasizes the pivotal and prolonged role of inflammatory reactions in the formation and development of coronary atherosclerotic lesions and the subsequently associated acute adverse cardiac events (Weber and Noels 2011). The development of AS is a long-term process, with corresponding lesions and symptoms that are not usually detected until they reach a moderate or advanced stage (Insull 2009). Therefore, early evaluation of arterial functions in patients with cardiovascular disease is critical in preventing acute adverse events and in the control of CHD. At present, the detection of arterial disease at an early stage is mainly dependent on the measurement of arterial elastic function parameters, including cardio-ankle vascular index (CAVI) and ankle brachial index (ABI) (Insull 2009). The latter two factors are examples of simple and noninvasive examinations that provide more detailed information for the early diagnosis and risk stratification of CHD. It has also been noted that the earliest pathological change in AS patients is the thickening of artery walls, with the media layers of the carotid arteries always being involved at the beginning of disease progression. Therefore, intima-media thicknesses (IMT) is an effective detection method for the clinical predictive indexes of CHD.

Haverkate et al. (1997) proposed that during the progression of AS, the concentration of serum hs-CRP increases gradually in part due to the aggravation produced by chronic inflammation and that the consequent vascular endothelial injury further promoted the release of cytokines, such as IL-6 and TNF-α, which in turn boosted the generation of CRP in the liver and activated immune reactions. Finally, thrombi develop as a consequence of the massive antibody and immune complexes that are deposited on the vascular endothelium (Singh et al. 2002). During the formation of thrombi, TNF-α is regarded as a major indicator of the instability of plaques, and the increase in TNF-α concentration is likely correlated with the aggravation of CHD (Nishimura et al. 2012). Therefore, the effective control of inflammatory factors will help to ameliorate the arterial lesions produced by AS.

The Huoxin formula was developed at the Shanghai University of Traditional Chinese Medicine to treat CHD, based on clinical experience. The formula contains *Asarum heterotropoides* (Fr.schmidt) var. *mandshuricum* (Maxim.) Kitag. (Aristolochiaceae), *Dalbergia odorifera* T. Chen (Leguminosae), *Panax notoginseng* (Burk.) F. H. Chen (Araliaceae) and *Astragalus membranaceus* (Fisch.) Bge. var. *mongholicus* (Bge.) Hsiao (Fabaceae) and other accessory materials, which have been reported to have cardioprotective (Xie et al. 2006; Zhang YG et al. 2008; Li J et al. 2011, 2012; Lim et al. 2013; Zhang et al. 2013), antithrombotic (Ku et al. 2013), anti-inflammatory (Zhang et al. 2014) and anti-apoptotic effects, as well as preventing low-density lipoprotein (LDL) oxidation (Chan et al. 2011; Zhu et al. 2013) in addition to its vasorelaxant activity (Yu and Kuo 1995).

In the present study, we added the Huoxin formula to the conventional CHD medications β-receptor blocker, statins, antiplatelet drugs with/without ACEI (ARB) drugs and other cardiovascular medications and analyzed the mechanisms of therapeutic benefits in addition to the effects on inflammatory-factors. We hypothesized that the Huoxin formula might have actions on arterial elasticity and inflammatory mediators.

## Materials and methods

### Study subjects

From October 2013 to December 2015, 58 patients diagnosed with CHD were included in the study from the outpatient clinic and the ward of the cardiovascular department in Shuguang Hospital, Shanghai University of Traditional Chinese Medicine. The ethical committee of Shuguang Hospital approved the study. Informed consent was obtained from all the subjects included in the study, which was carried out in accordance with approved guidelines. The clinical registration number of the study is ChiCTR-TRC-13004040, and the date of registration 19 December 2013.

### Diagnostic criteria

The medical diagnosis was based on ‘The diagnostic and therapeutic guidelines of chronic stable angina pectoris’ published by the Chinese Society of Cardiology and the editorial board of the Chinese Journal of Cardiology in 2007 (Chinese Society of Cardiology and Editorial Board 2007), which are basically the same as for conventional cardiovascular drugs. Characteristic location and nature of chest pain, with small duration times induced by labour or emotional agitation lasting ≤10 min, with no obvious abnormalities found during physical examination. ECG exercise tests revealed typical angina pectoris attacks during exercise with horizontal or downward sloping ST segment depression of more than 1 mm during or after exercise (60–80 ms) after the J point or patient blood pressure dropped during exercise.

*Inclusion criteria:* Diagnosis of stable angina pectoris and aged between 18 and 75 years, with no restrictions on gender.

*Exclusion criteria:* Patients with serious complications were excluded including: severe primary diseases of the cardiovascular system, liver and haemopoietic system; serious diabetic complications or hypertensive complications; pronounced infections or disturbances in electrolyte balance; mental disorders. Women who were pregnant or breastfeeding were excluded. Those recognized as inappropriate participants by our research centre for various reasons were also excluded.

### Treatment protocols

According to ‘The diagnostic and therapeutic guidelines of chronic stable angina pectoris’, all the patients included in the study were treated with conventional CHD medicines, mainly including β-receptor blocker, statins, antiplatelet drugs with/without ACEI (ARB) drugs, and fewer nitrates, aspirin, enteric-coated tablets as well as angiotensin-converting enzyme inhibitors when necessary. Patients were randomized into an intervention group (*n* = 30) and a control group (*n* = 28). The patients in the control group were treated only with the conventional CHD medicines, while the patients in the intervention group were additionally medicated with the Huoxin formula.

### Huoxin formula

The Huoxin formula consists of a mixture from roots of *A. heterotropoides* (Fr. Schmidt) var. *mandshuricum* (Maxim.) Kitag. (Aristolochiaceae), *D. odorifera* T. Chen (Leguminosae), *P. notoginseng* (Burk.) F. H. Chen (Araliaceae) and *Ast. membranaceus* (Fisch.) Bge. var. *mongholicus* (Bge.) Hsiao (Fabaceae) (Table 1).

**Table 1. t0001:** Ingredients of the Huoxin formula.

	*Astragalus membranaceus* (Fisch.) Bge. var. *mongholicus* (Bge.) Hsiao (Fabaceae)	*Panax notoginseng* (Burk.) F. H. Chen (Araliaceae)	*Dalbergia odorifera* T. Chen (Leguminosae)	*Asarum heterotropoides* (Fr.schmidt) var. *mandshuricum (*Maxim.) Kitag. (Aristolochiaceae)
Plant parts	Root	Root	Root	Root
Amount of plant material before and after extraction	30 g (4.5 g)	3 g (no extract)	6 g (0.5 g)	3 g (0.5 g)
Application mixture (twice per day)	2 × 4.5 g	2 × 3.0 g	2 × 0.5 g	2 × 0.5 g
Chemical composition	Polysaccharides [AG-1 (astragalus glucan-1), AG-2 (astragalus glucan-2), AH-1 (astragalus heteroglycan-1), AH-2 (astragalus heteroglycan-2), d-glucose, d-galactose, and l-araban], flavonoids [7, 3-dimercapto-4, 1-methoxyisoflavone, 3-dimercapto-7, 4, 1-methoxyisoflavone, catycosin, kumatakenin and fomononetin], numerous amino acids [daucosterol, choline, betaine, folic acid, nicotinamide, and linoleic acid], trace elements and various other components, such as astragalus saponin I–II, astragalosides I–IV, soyasapogenoside, β-sterol, lupeol, hexanol, palmitic acid, 6-*o*-β-d-pyranoglucose, 3-*o*-β-d-xylopyranose and carotenol	Saponins (notoginsenosides A–E, G–N, U, R1–R4, R6 and R7; ginsenosides Rb1, Rb2, Rd, Re, Rg1, Rg2, Rh1, Rh4 and U; 20-*O*-glucoginsenoside Rf; dannar-20(22)-ene-3β,12β,25-triol-6-*O*-β-d-glucopyranoside; gypenoside XVII.), favonosides (quercetin), polysaccarides (sanchian A) and amino acids, Others are notoginsenic acid β-sophoroside, dencichine, β-sitosterol, daucosterol, panaxydol and panaxynol	Volatile oils (α-pinene, camphene, β-pinene, myrcene, sabinene, limonene, 1,8-cineole, *p*-cymene, γ-terpinene, terpinolene, borneol, esTCMLIBagole, 2-isopropyl-5-methylanisole, 3, 5-dimetho-xytoluene, safrole, methyl eugenol, asaricin, myristicin, elemicin)	Volatile oil (β-bisabolene, *trans*-β-farnesene, *trans*-nerolidol), liquiritigenin, isoliquiritigenin, formoononetin, medicarpin, pterocarpin, styrene

The ready-to-use herbal granules (distributed by the Shuguang pharmacy in the Shanghai University of Traditional Chinese Medicine and purchased from Jiangyin Tianjiang Pharmaceuticals Co., Ltd., Jiangyin, China), were dissolved at home and taken by each patient twice a day for 3 months. Each medication consisted of granules from packages with single herbs, which have been poured into a cup and solved in 150 mL of warm water. The producer guaranteed high quality according to the Good Agricultural Practice of Medicinal Plants and Animals advanced quality control, with detection technologies including fingerprint ([Fig F0001] Supplementary Figure 1 ) and specific chromatograms as well as high performance liquid chromatography-mass spectrometry in order to maintain constant quality of mixture batches ([Fig F0002] Supplementary Figure 2 ; [Table t0001] Supplementary Table 1 ).

**Figure 1. F0001:**
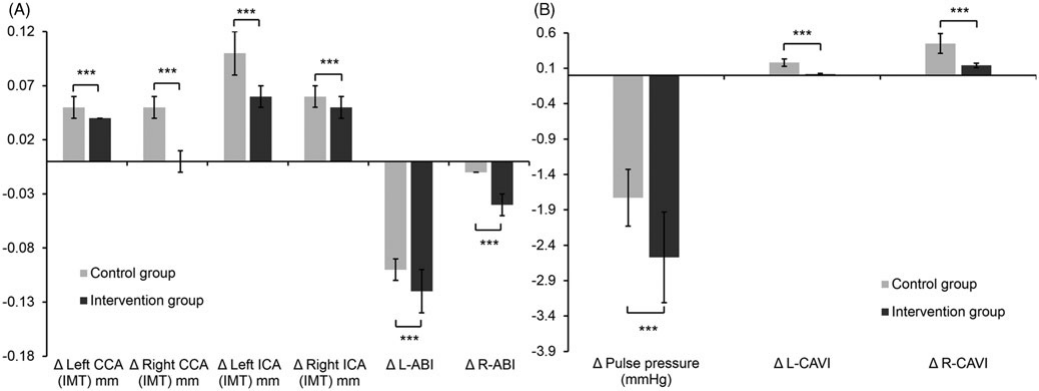
Differences of (A) left and right CCA and ICA IMTs as well as ABIs and (B) pulse pressure and left and right CAVI values in the intervention and control groups before and 3 months after the initiation of therapy. ***p < 0.001.

**Figure 2. F0002:**
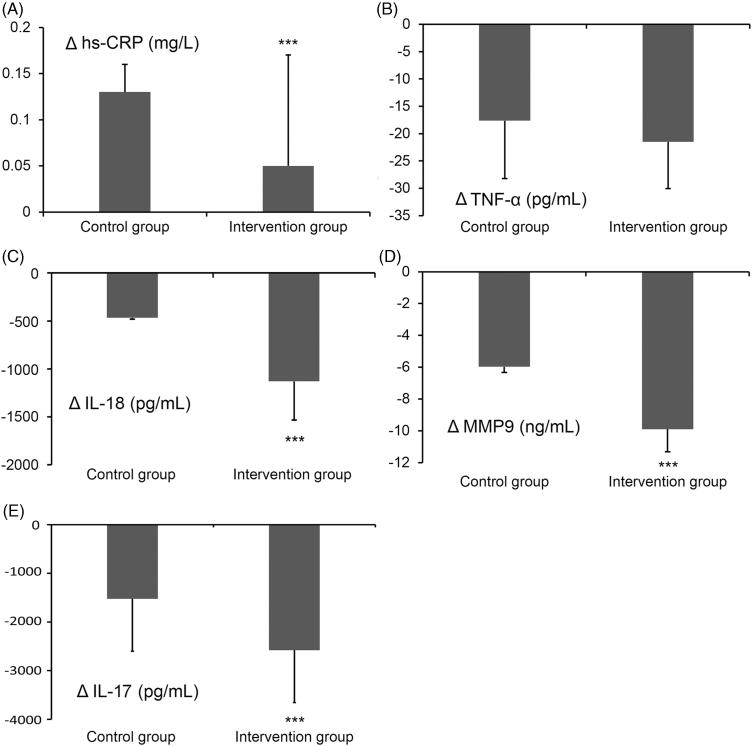
Differences of inflammatory factor serum concentrations in the control and intervention groups before and 3 months after the initiation of therapy. (A) Increase of hs-CRP, (B) decrease of TNF-α, (C) decrease of IL-18, (D) decrease of MMP9 and (E) decrease of IL-17. ***p < 0.001 between the control and intervention group.

### Clinical indices and measuring methods

Safety indexes (blood, urine, liver and kidney functions, blood lipids, blood glucose, glycosylated haemoglobin, UCG, ECG) and effect indexes (hs-CRP, IL-18, IL-17, TNF-α, MMP-9, CAVI, ABI, IMT, pulse pressure) were measured before the treatment, as well as 3 months after the initiation of therapy.

### Serum collection and measurement methods

We collected 4 mL blood from the cubital veins of each fasting patient in the morning, and centrifuged the samples for 10 min at 3000 rpm. The separated serum was stored in a freezer at −70 °C. Factors such as hs-CRP, IL-18, IL-17, TNF-α and MMP-9 were measured using ELISA (eBioscience, San Diego, CA).

### Arterial function analysis

#### Pulse pressure

Pulse pressure (mmHg) = systolic blood pressure − diastolic blood pressure (mmHg). After resting the patients for 5 min, a mercury sphygmomanometer was used to measure the blood pressure in the right upper arm with the patient lying on a bed. The mercury column was brought down at a rate of 2 mmHg/s, and 2 min after the first measurement was completed, a second reading was taken and the mean blood pressure calculated. Systolic and diastolic blood pressure was recorded when the first and fifth Korotkoff sounds were separately detected.

### CAVI and ABI determinations

These two indexes were measured with a VaSeraTM VS-1000, which electronically measures blood pressure and the pulse (Beijing Electronic Medical Instrument Co., Ltd, Futian, Beijing, China). When patients were resting in a prone position on a bed, the pressure cuff and sensor was applied. The pressure cuffs were set at the same positions as when measuring blood pressure and small cushions were placed around the elbows and heels of patients. The cuffs were kept at the same level as the heart in order to keep the pulse wave stable. Two ECG electrodes were attached to the wrists to record waveforms from lead I. A phonocardiogram (PCG) sensor was added to the right knee with the gasbag pointing at the centre of the back of the knee. The strap was adjusted until the green light on the sensor was illuminated (on). Then, we connected and fixed Velcro beside the kneecap, and made sure the pulse wave was clear and stable. Then the START button was pressed to initiate the measurements, with the PCG and stable pulse waves displayed on a monitor. CAVI and ABI reports were automatically printed when the measurements were completed.

### Common and internal carotid artery IMTs

IMTs were measured using a HP Sonos 5500 (Hewlett Packard) ultrasound instrument at a frequency of 7.0–10 MHz. Patients were placed in the supine position with a head deviation of 45 degrees to the side, with the neck fully exposed. All measurements of the anterior and posterior walls of the CCA, 1 cm proximal to the bilateral carotid branch and in the ICA were performed by the same senior surgeon and the average value of the measurements computed; local plaques were not included.

### Statistical analysis

SPSS Statistics for Windows (Version 17.0., SPSS Inc., Chicago, IL) was used to perform statistical analyses with *p* < 0.05 considered to be statistically significant. Measurements of normally distributed data are presented as the mean ± standard deviation (x ± SD), while data with a skewed distribution are presented as the median, minimum and maximum values. Enumeration data was described by constituent ratios. A chi-squared test was performed to compare the rates of two samples and variance analysis was conducted to compare the repeated measurements in the follow-up (baseline data was set as the concomitant variable).

## Results

There were no significant differences in gender, age, course of disease, or increased incidences of complications between patients in the two groups (*p* > 0.05), indicating the comparability of the therapeutic effects of the two different treatments (Table 2). In addition, in both groups, the blood glucose and lipid concentrations did not significantly change during the treatment period (Table 3).

**Table 2.** Baseline characteristics of the patients.

	Intervention group (*n* = 30)	Control group (*n* = 28)	*p*-value
Gender			1.000
Male	20	18	
Female	10	10	
Age (years)	66.8 ± 7.6	66.5 ± 9.7	0.896
Course of disease (months)	5.4 ± 1.3	5.1 ± 1.4	0.401
Complication			0.355
Hypertension	19	18	
Hyperlipemia	17	20	
Diabetes	6	10	

**Table 3.** Blood lipid and glucose levels before and after treatments.

	Intervention group (*n* = 30)			Control group (*n* = 28)		
	Before treatment	After treatment	*p*-value	Before treatment	After treatment	*p*-value
Triglyceride (mmol/L)	2.05 ± 1.38	1.60 ± 1.01	0.155	2.08 ± 1.04	2.04 ± 1.21	0.895
Cholesterol (mmol/L)	4.60 ± 1.28	4.52 ± 1.22	0.805	5.44 ± 1.82	5.36 ± 1.77	0.868
HDL (mmol/L)	1.09 ± 0.26	1.13 ± 0.28	0.569	1.05 ± 0.26	1.04 ± 0.31	0.896
LDL (mmol/L)	2.49 ± 0.94	2.53 ± 1.04	0.876	3.15 ± 1.23	3.15 ± 1.16	1.000
Blood glucose (mmol/L)	5.94 ± 1.25	6.11 ± 1.96	0.690	6.51 ± 3.02	5.67 ± 1.56	0.196
HbA1c (%)	6.14 ± 1.30	5.97 ± 0.81	0.546	6.25 ± 1.11	6.17 ± 1.35	0.810

### Comparison of changes in arterial functional indexes 3 months after the initiation of treatment

Comparison of baseline arterial functional indexes revealed that apart from R-ABI values all indexes did not significantly differ in the intervention and control groups before the initiation of therapy (Table 4).

**Table 4.** Comparison of baseline arterial functional indexes in two groups.

	Intervention group (*n* = 30)	Control group (*n* = 28)	*p*-value
Left common carotid artery (IMT) mm	0.90 ± 0.16	0.80 ± 0.12	0.078
Left internal carotid artery (IMT) mm	0.68 ± 0.19	0.55 ± 0.17	0.065
Right common carotid artery (IMT) mm	0.93 ± 0.15	0.80 ± 0.15	0.602
Right internal carotid artery (IMT) mm	0.68 ± 0.19	0.64 ± 0.21	0.608
R-CAVI	8.23 ± 1.25	7.54 ± 2.14	0.338
L-CAVI	8.45 ± 1.72	8.45 ± 1.65	0.997
R-ABI	1.05 ± 0.15	1.13 ± 0.02	0.040
L-ABI	1.21 ± 0.1	1.22 ± 0.09	0.690
Pulse pressure (mmHg)	52.00 ± 10.37	52.91 ± 7.18	0.634

However, compared to the baseline data before treatment, IMT in the left and right CCAs, and in the ICAs were significantly less increased in the intervention group than in the control group after 3 months of therapy (Figure 1(A)). Notably, decreases of ABI and CAVI on both sides were more marked in the intervention group. Thus, the progression of coronary stenosis slowed down more significantly in patients treated with a combination of Chinese and conventional CHD medicines (Figure 1(A,B)).

### Comparison of changes in serum inflammatory factors 3 months after the initiation of treatment

As shown in Table 5, baseline serum concentrations of the indicated inflammation indicators did not significantly differ in the intervention and control groups. However, after 3 months of therapy, the expression changes of hs-CRP were significantly less increased (*p* < 0.001) in the intervention group. The reductions of IL-18 (*p* < 0.001), IL-17 (*p* < 0.001) and MMP9 (*p* < 0.001) as well as TNF-α levels were greater in the intervention group, indicating that the combinatory use of traditional chinese medicine was able to suppress systemic inflammatory reactions (Figure 2).

**Table 5.** Comparison of the baseline serum inflammatory factors in two groups.

	Intervention group (*n* = 30)	Control group (*n* = 28)	*p*-value
hs-CRP (mg/L)	1.79 ± 1.25	2.26 ± 1.22	0.110
TNF-α (pg/mL)	52.88 ± 0.86	41.63 ± 23.37	0.237
IL-18 (pg/mL)	4244.14 ± 1939.31	4107.87 ± 1863.95	0.850
MMP9 (ng/mL)	154.23 ± 19.36	143.03 ± 28.31	0.196
IL-17 (pg/L)	6023.48 ± 1986.12	5880.35 ± 1598.26	0.765

### Correlations between arterial functional measurements and the concentrations of serum inflammatory factors

Based on a correlation analysis, we found that there were various significant intra and inter correlations between CCA IMTs, ICA IMTs and left ABI as well as between CAVIs and ABIs, whereas pulse pressure correlated with left and right ABI (Table 6), which indicates that AS is a systemic disorder. Additionally, TNF-α correlated with the IMT of the right CCA (r = 0.392, *p* = 0.043) as well as with R-CAVI *(r* = 0.526, *p* = 0.037) and L-CAVI (*r* = 0.619, *p* = 0.011). IL-18 correlated with left ICA IMT (*r* = 0.594, *p* = 0.007) and pulse pressure (*r* = 0.606, *p* = 0.004) (Table 6), whereas IL-17 correlated with left ICA (*r* = 0.575, *p* = 0.006) indicating that inflammatory factors contribute to blood vessel obstructions.

**Table 6.** Correlation analysis between the arterial functional measurements and the concentrations of serum inflammatory factors in patients from the intervention group.

Correlation	*r*	*p*-value
Left CCA (IMT) mm		
vs. Right CCA (IMT) mm	0.811	<0.001
Left ICA (IMT) mm		
vs. Right CCA (IMT) mm	0.593	0.006
vs. Right ICA (IMT) mm	0.905	<0.001
vs. hs-CRP (mg/L)	0.581	0.009
vs. IL-18 (pg/mL)	0.594	0.007
vs. IL-17(pg/L)	0.575	0.006
Right CCA (IMT) mm		
vs. Right ICA (IMT) mm	0.501	0.021
vs. L-ABI	−0.419	0.017
vs. hs-CRP (mg/L)	0.450	0.041
vs. TNF-α (pg/mL)	0.392	0.043
Right ICA (IMT) mm		
vs. hs-CRP (mg/L)	0.477	0.029
R-CAVI		
vs. L-CAVI	0.708	0.001
vs. R-ABI	0.393	0.026
vs. TNF-α (pg/mL)	0.526	0.037
L-CAVI		
vs. TNF-α (pg/mL)	0.619	0.011
R-ABI		
vs. L-ABI	0.767	<0.001
vs. Pulse pressure (mmHg)	−0.552	0.001
L-ABI		
vs. Pulse pressure (mmHg)	−0.745	<0.001
Pulse pressure (mmHg)		
vs. IL-18 (pg/mL)	0.606	0.004

Further correlation analyses revealed that IL-18 was correlated with IL-17 (*r* = 0.328, *p* = 0.021) (Table 7).

**Table 7.** Correlation analysis between the concentrations of serum inflammatory factors in patients from the intervention group.

	hs-CRP (mg/L)	TNF-α (pg/ml)	IL-18 (pg/mL)	MMP9 (ng/mL)	IL-17 (pg/L)
hs-CRP r	1.000	0.001	−0.094	0.184	−0.076
*p*-value		ns	ns	ns	ns
TNF-α r		1.000	0.006	0.377	0.036
*p*-value			ns	ns	ns
IL-18 r			1.000	0.137	**0.328**
*p*-value				ns	**0.021**
MMP9 r				1.000	0.247
*p*-value					ns
IL-17 (pg/L)					1.000

*Bold values indicates: IL-18 vs. IL-17: r = 0.328, *p* = 0.021.

According to the literature, there is no report that *Radix astragali, Panax, D. odorifera* and *Asarum* cause liver injury (Xiao and Zhang 2017) and there were no reported adverse events related to the Huoxin formula in the present study.

## Discussion

CHD is caused by coronary atherosclerotic stenosis or occlusion of the lumen, resulting in myocardial ischemia and hypoxia. The risk factors include being a male, obesity, smoking, hypertension, diabetes mellitus, dyslipidemia and other factors (Cobble 2014). Although the disease is more common in middle-aged and elderly people over the age of 40, in recent years, with a change of Chinese people to a western style diet, the age of onset showed a trend towards younger patients, since the degree of smoking and obesity in young people is gradually increasing. In particular, IL-17 released by visceral adipose tissue has been proposed to be a factor for early AS in obese people by inducing eotaxin secretion of smooth muscle cells in atheromatosus vessels (Tarantino et al. 2014). In our study, circulating IL-17 levels were significantly reduced in the intervention group, which indicated that the treatment might have had an effect on lesion cell activities and inflammation in atherosclerotic lesions (de Boer et al. 2010; van Bruggen and Ouyang 2014).

In the present study, ABI, CAVI and pulse pressure were significantly improved in patients treated with Huoxin formula for 3 months. These findings support the role of Huoxin formula in promoting the coronary circulation, reducing platelet adhesion, and preventing medial thickening (Hu 2009). In previous studies, *Ast. membranaceus* (astragaloside IV) and *P. notoginseng* (notoginsenoside R1) have been shown to protect against cardiac hypertrophy (Chen et al. 2015), and ginsenosides were reported to improve the blood circulation, enhance vasomotor tone and regulate the lipid profile, mainly through inhibition of reactive oxygen species production (Chan et al. 2002; Lee et al. 2014). *Astragalus membranaceus* polysaccharides have been shown to reduce cohesion between human cardiac microvascular endothelial cells and polymorphonuclear leukocytes by inhibiting their adhesion molecule expression and by downregulation of p38MAPK signalling (Zhu et al. 2013), as well as effectively protecting against LDL oxidation (Chan et al. 2011). Since free radicals in the endothelium can lead to oxidized LDL, which in turn activates platelet aggregation and MAP kinase activity with concomitant inflammation signalling during atherosclerotic plaque formation (Singh et al. 2002), the reductions of CAVI and IMT increases may be partly attributed to the activities of *Ast. membranaceu* (Radix astragali) and *P. notooginseng* (Pseudoginseng). We also analyzed the concentrations of multiple serum inflammatory factors before and after combinatory therapy. The concentration of IL-18 was positively correlated with the incidence of cardiovascular events (Blankenberg et al. 2003; Furtado et al. 2009) and in patients diagnosed with unstable angina pectoris; the higher the concentration of IL-18, the greater was the incidence of adverse cardiovascular events (Li et al. 2014). Therefore, it has been proposed that the reduction of adverse cardiovascular events can be attributed to a decrease in the concentration of IL-18 (Kuo et al. 2008). The decrease of IL-18 serum concentration in the intervention group was 2.4 times that of the control group (Figure 2). Increased MMP-9 serum levels have been shown to occur in congestive heart failure patients (Wilson et al. 2002) and in a previous study, it was reported that IL-18 is a strong inducer of MMP-9 release (Nold et al. 2003). Our data showed that serum concentrations of IL-18 and MMP-9 were significantly reduced in the intervention group compared to the control group, which is in accordance with a previous study in which *P. notoginseng* administration reduced IL-18, IL-1β and MMP-2 and 9 serum concentrations in a rat AS model (Zhang et al. 2008). In addition *P. notoginseng* function as an anti-inflammatory agent through directly targeting Th17 cell-mediated immune response (Wei et al. 2017).

Also, the accessory *D. odorifera* has been shown to exert anti-inflammatory activity by activating heme oxygenase-1 (HO-1) in macrophages (Lee et al. 2009, 2014) and also to increase coronary blood flow (Sugiyama et al. 2002).

In summary, we suggest that the positive effect on coronary vessel integrity of the Huoxin formula was related to the attenuation of inflammatory factors. However, the sample size analyzed in our study was relatively small, which is a limitation of the present study.

Vascular cell adhesion molecule 1 (VCAM-1) and intercellular adhesion molecule 1 (ICAM-1) are expressed by endothelial cells following cytokine signalling and bind lymphocytes through cell surface adhesion molecules (Davies et al. 1993). ICAM-1 has been reported to be involved in early AS formation and angina (Hwang et al. 1997; Ridker 1998; Jude et al. 2002; Luc et al. 2003; Fotis et al. 2012) and anti-adhesion therapies have been proposed as a novel means to prevent cardiovascular disease progression (Ridker et al. 1998). In previous studies *Asarum* has been found to suppress the expression of VCAM-1 and ICAM-1 (Zhang et al. 2006; Lee et al. 2014), thereby protecting blood vessels from inflammatory processes.

## Conclusions

Huoxin formula as an adjunct to conventional stable angina pectoris medication slowed down IMT in the common and ICAs, improved CAVI and ABI values as well as pulse pressure significantly better than conventional CHD medicine alone, which was related with changes of inflammatory factor activities. Thus, the progression of coronary stenosis was significantly slowed down in patients treated with adjunctive Huoxin formula.

## Supplementary Material

Supplementary_files.docx
