# Supplementary material for: Effects of huoxin formula on the arterial functions of patients with coronary heart disease
Source: Pharm Biol. 2019 Jun 14;57(1):13–20. doi: 10.1080/13880209.2018.1561726 (PMC6586089; doi:10.1080/13880209.2018.1561726)
Supplement: Supplementary_files.docx [file IPHB_A_1561726_SM4985.docx]

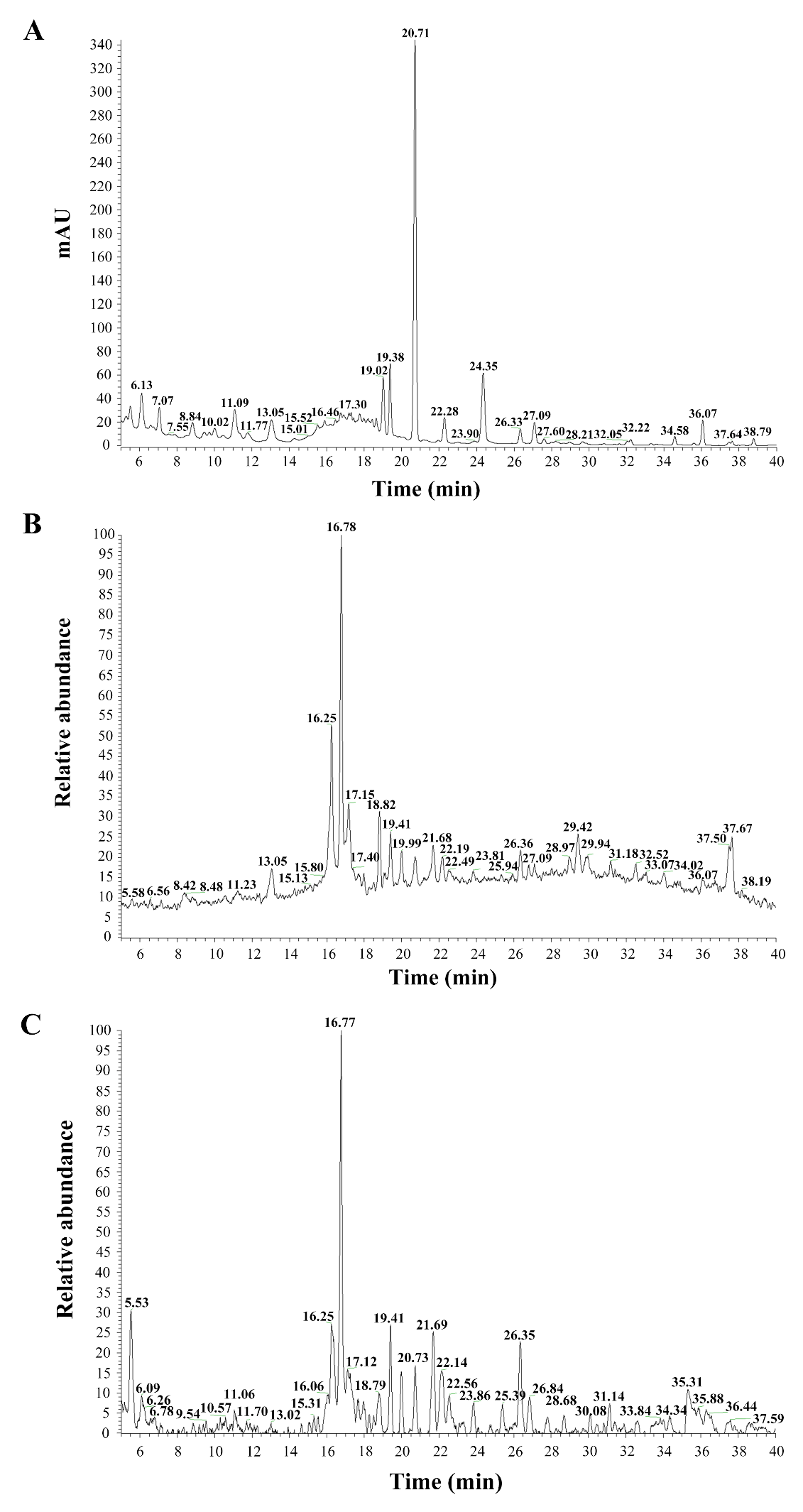


**Supplementary Figure 1.** Fingerprint (A) UV-detector liquid chromatogram, (B) Total ion chromatograms of positive ion mode mass spectrum and (C) Total ion chromatograms of negative ion mode mass spectrum

**
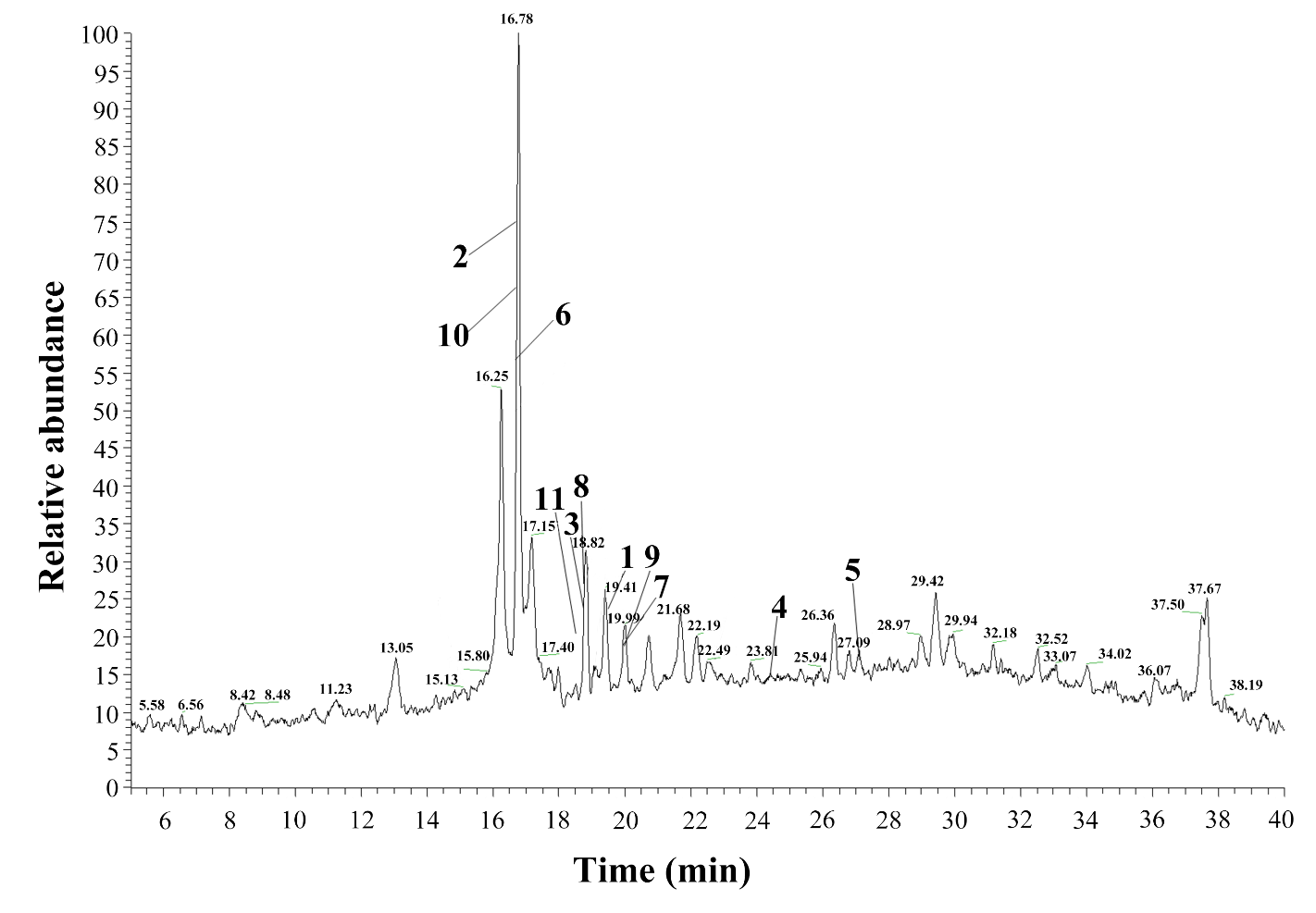
**

**Supplementary Figure 2.** HPLC-MS of Huoxin formula

**Supplementary Table 1. The mass spectrum identification of Huoxin formula compounds**

| Peak No. | t_R_/min | Molecular formula | Ion mode | Molecular ion peak | | Error (×10^-6^) | Fragment ion (Arrange by abundance) | Compound name | Source |
| --- | --- | --- | --- | --- | --- | --- | --- | --- | --- |
|  |  |  |  | Theoretical value | Measured value |  |  |  |  |
| 1 | 19.41 | C_16_H_12_O_5_ | [M-H]^-^ | 283.0611 | 283.0612 | 0.37 | 268.0378, 283.0614, 240.0425, 135.0073 | Wogonin | *Radix astragali* |
| 2 | 16.75 | C_47_H_78_O_19_ | [M-H]^-^ | 945.5064 | 945.5067 | 0.98 | 161.0444, 475.3797, 637.4329, 179.0553, 143.0339, 267.0868, 391.2872 | Astragaloside V | *Radix astragali* |
| 3 | 18.50 | C_16_H_12_O_5_ | [M+H]^+^ | 285.0757 | 285.0768 | 3.83 | 285.0767, 270.0531, 253.0503, 225.0549, 137.0237 | Calycosin | *Radix astragali* |
| 4 | 27.08 | C_17_H_16_O_5_ | [M+H]^+^ | 301.1070 | 301.1082 | 3.97 | 167.0708, 147.0444, 191.0709, 269.0814, 301.1078, 207.0661 | 3-Hydroxy-9,10-dimethoxyptercarpan | *Radix astragali* |
| 5 | 22.47 | C_15_H_10_O_5_ | [M-H]^-^ | 269.0455 | 269.0456 | 4.59 | 269.0457, 151.0028 | Baicalein | *Radix astragali* |
| 6 | 16.77 | C_42_H_72_O_14_ | [M-H]^-^ | 799.4849 | 799.4838 | -1.38 | 475.3796, 637.4319, 391.2857 | Ginsenoside Rg1 | *Panax notoginseng* |
| 7 | 19.99 | C_41_H_70_O_13_ | [M-H]^-^ | 769.4743 | 769.4741 | -0.26 | 475.3797, 161.0444, 637.4338, 191.0554, 391.2864, 318.0181 | Notoginsenoside R2 | *Panax notoginseng* |
| 8 | 18.83 | C_54_H_92_O_23_ | [M-H]^-^ | 1107.5956 | 1107.5961 | 0.45 | 621.4370, 459.3846, 783.4906, 945.5441 | Ginsenoside Rb1 | *Panax notoginseng* |
| 9 | 19.79 | C_53_H_90_O_22_ | [M-H]^-^ | 1077.5850 | 1077.5847 | -0.28 | 621.4378, 459.3858, 783.4913, 945.5443, 375.0625 | Ginsenoside Rb2 | *Panax notoginseng* |
| 10 | 16.25 | C_47_H_80_O_18_ | [M-H]^-^ | 931.5271 | 931.5277 | 0.64 | 475.3797, 637.4331, 391.2867 | Notoginsenoside R1 | *Panax notoginseng* |
| 11 | 18.45 | C_20_H_18_O_6_ | [M-H]^-^ | 353.1030 | 353,1032 | 3.69 |  | Asarinin | *Asarum* |

Note. The main components of *Dalbergia odorifera* are flavonoids and volatile oils, which are not been characterized ingredients and therefore quantitative and qualitative analyses are not possible.
